# *Shigella flexneri* vaccine development: Oral administration of peptides derived from the 49.8 kDa pili protein subunit activates the intestinal immune response in mice

**DOI:** 10.14202/vetworld.2022.281-287

**Published:** 2022-02-11

**Authors:** Khoirul Anam, Agustina Tri Endharti, Sri Poeranto, Hidayat Sujuti, Dwi Yuni Nur Hidayati, Sumarno Reto Prawiro

**Affiliations:** 1 Doctoral Program in Medical Science, Faculty of Medicine, Universitas Brawijaya, Malang, Indonesia; 2 Study Program of Medical Laboratory Technology, Institute of Health and Science Technology Wiyata Husada, Samarinda, Indonesia; 3 Department of Parasitology, Faculty of Medicine, Universitas Brawijaya, Malang, Indonesia; 4 Department of Biochemistry, Faculty of Medicine, Universitas Brawijaya, Malang, Indonesia; 5 Department of Clinical Microbiology, Faculty of Medicine, Universitas Brawijaya, Malang, Indonesia

**Keywords:** epitope, immune response, oral, *Shigella flexneri*, shigellosis

## Abstract

**Background and Aim:**

The morbidity and mortality of *Shigella* infections remain a global challenge. Epitope-based vaccine development is an emerging strategy to prevent bacterial invasion. This study aimed to identify the ability of the 49.8 kDa pili subunit adhesin protein epitope of *Shigella flexneri* to induce an intestinal immune response in mice.

**Materials and Methods:**

Thirty adult male Balb/c mice were divided into a control group, cholera toxin B subunit (CTB) group, CTB+QSSTGTNSQSDLDS (pep_1) group, CTB+DTTITKAETKTVTKNQVVDTPVTTDAAK (pep_2) group, and CTB+ ATLGATLNRLDFNVNNK (pep_3). We performed immunization by orally administering 50 μg of antigen and 50 μl of adjuvant once a week over 4 weeks. We assessed the cellular immune response by quantifying T helper 2 (Th2) and Th17 using flow cytometry. In addition, we assessed the humoral immune response by quantifying interleukin (IL-4), IL-17, secretory immunoglobulin A (sIgA), and β-defensin using enzyme-linked immunoassay. Statistical analysis was performed using one-way analysis of variance and Kruskal–Wallis test.

**Results:**

Peptide oral immunization increases the cellular immune response as reflected by the increase of Th2 (p=0.019) and Th17 (p=0.004) cell counts, particularly in the CTB_pep_1 group. Humoral immune response activation was demonstrated by increased IL-4 levels, especially in the CTB+pep_3 group (p=0.000). The IL-17 level was increased significantly in the CTB+pep_1 group (p=0.042). The mucosal immune response was demonstrated by the sIgA levels increase in the CTB+pep_3 group (p=0.042) and the β-defensin protein levels (p=0.000).

**Conclusion:**

All selected peptides activated the cellular and humoral immune responses in the intestine of mice. Further studies are necessary to optimize antigen delivery and evaluate whether the neutralizing properties of these peptides allow them to prevent bacterial infection.

## Introduction

Shigellosis is an acute gastrointestinal infectious disease characterized by bloody stool diarrhea. Its mortality and morbidity rates are particularly high in low- and middle-income economy countries. It is highly prevalent in children under 5 years, particularly in poor sanitary conditions and overcrowded populations [1]. One of the pathogenic causes of shigellosis is *Shigella flexneri* species, and this species is frequently recorded in shigellosis cases worldwide [2-4]. The disease burden and antimicrobial resistance of *S. flexneri* infection remain global challenges and overcoming them require developing new strategies [5,6].

*Shigella* invasion is initiated by bacterial adhesion to the enteric epithelial lining. Therefore, the pili portion of *Shigella* is crucial for bacterial host infection. Sumarno *et al*. [7] reported that the pili of *Shigella dysenteriae* contain a hemagglutinin protein weighing 49.8 kDa that acts as an adhesin [8]. Other *Shigella* species such as *S. flexneri*, *Shigella sonnei*, and *Shigella* *boydii* have similar pili proteins [9]. The importance of pili proteins was demonstrated by administering the 37.7 kDa pili protein subunit *Vibrio cholerae* 01, which stimulated the mucosal immune response by increasing soluble immunoglobulin A (sIgA) levels [10]. Orally administering the 49.8 kDa pili protein subunit of *S. dysenteriae* yielded a similar response in mice [11].

T helper 2 (Th2) and Th17 cells promote the mucosal immune response toward *Shigella* infection. The Th2 cells mediate the humoral immune response by secreting interleukin 4 (IL-4), IL-5, IL-6, and IL-13 [12]. The immunoglobulin A (IgA) immune response induced by Th2 cells inhibits the bacterial attachment to epithelial cells [13]. Moreover, Th17 cells have a key role in the pathogenesis of autoimmune and inflammatory diseases [14]. The differentiated Th17 cells secrete the cytokines IL-17 and IL-22. These cytokines promote the release of secretory IgA (sIgA) in the intestinal lumen and contribute to antimicrobial proteins secretion [15,16]. Antimicrobial peptides (AMPs) also play crucial roles in the adaptive immune system and pathogen elimination [17].

Despite the role of pili proteins in humoral immune response induction, *S. flexneri* immunization combined with cholera toxin B subunit (CTB) and Sumbawa horse milk administration increases sIgA and β-defensin levels [18]. The developing countries would greatly benefit from efficacious and safe vaccines against *Shigella*. However, such vaccines require further development [3]. The current vaccine candidates are limited to attenuated strain vaccines, conjugate vaccines, subunit vaccines (Invaplex, OMP, T3SS), and combination vaccines (*Shigella*-ETEC and *Shigella*-Salmonella) [19]. The current efforts toward vaccines or early detection diagnostic tests using pili proteins are promising [20]. Identifying the adhesin protein sequences revealed protein epitopes potentially useful for vaccine development. Pore *et al*. [21] analyzed the amino acid sequence of the 34 kDa outer membrane protein (OmpA) of *S. flexneri*. Similarly, Sharma *et al*. [22] performed epitope prediction modeling on the outer membrane proteins (OMPs) of *S. flexneri* 2a.

The chemical stability, easy production, and minimal infection potential of peptides make them attractive vaccine candidates. The previous peptide vaccines have been developed and approach the clinical stage. Several studies demonstrated the effectiveness of epitope-based vaccine design in stimulating long-term antibody production and protective immunity against *V. cholera* [23-25]. In addition, our previous study demonstrated the epitope sequence of the 49.8 kDa pili protein from *S. flexneri*-induced antipeptide serum antibodies and prevented leaking in the enterocyte of mice [26].

This study aimed to document the antigenic properties of the epitopes of the 49.8 kDa *S. flexneri* pili protein by assessing their ability to induce cellular and humoral immune responses in the intestine of mice. This study should provide fundamental data for the development of epitope-based vaccines against *S. flexneri* as part of the effort to overcome the shigellosis infection issues.

## Materials and Methods

### Ethical approval

Adult male Balb/c mice were obtained and housed in Experimental Animal Laboratory, Faculty of Medicine, Universitas Brawijaya. The animals were kept under maintained humidity, temperature, and regular dark-light cycle. All animals had free access to drink and standard laboratory food [27]. All procedures were previously approved by Research Ethics Committee, Universitas Brawijaya (no.1192-KEP-UB).

### Study period and location

This study was conducted from January to March 2021 at Experimental Animal Laboratory, Clinical Parasitology Laboratory, and Biomedical Central Laboratory, Faculty of Medicine, Universitas Brawijaya.

### Experimental design

We separated 30 adult male Balb/C mice (6-8 weeks old) into three groups (each group had 10 mice); control group, CTB group, CTB+QSSTGTNSQSDLDS (pep_1) group, CTB+DTTITKAETKTVTKNQVVDTPVTTDAAK (pep_2) group, and CTB+ATLGATLNRLDFNVNNK (pep_3). We used CTB as an adjuvant. We performed immunization by orally administering 50 μg of peptide in 50 μl of adjuvant once a week for 4 weeks. We sacrificed the mice 1 week after the last immunization [28]. As antigens, we used synthetic peptides from the 49.8 kDa *S. flexneri* pili protein named pep_1, pep_2, and pep_3 (Apical Scientific Sdn. Bhd., Malaysia).

### Peptide sequencing and modeling

Isolated protein from *S. flexneri* pili was denatured in 100°C of Laemmli buffer and electrophoresed in SDS-PAGE on 4% gel. Coomassie brilliant blue was applied to visualized protein bands. The protein band at 49,8 kDa was excised and then dried and sent it for sequencing analysis at Proteomic International (Australia) according to the in-gel digestion method and spectral analysis using an electrospray ionization mass spectrometer (Agilent 1260 Infinity HPLC system, USA) paired with an Agilent 6540 mass spectrometer. Data were analyzed using Mascot software (Matrix Science, USA) with Ludwig NR database [29]. We modeled the secondary protein structure of the peptides using the PEP-FOLD3 server (http://mobyle.rpbs.univ-paris-diderot.fr/cgi-bin/portal.py#forms: PEP-FOLD3).

### Molecular analysis

#### Flow cytometry

We counted Th2 andTh17 cells in intestinal mesenteric tissue using a flow cytometry apparatus (BD FACS Melody, USA). We performed Th2 and Th17 double staining using FITC-labeled anti-mouse CD4 antibody (BioLegend, San Diego, CA, USA) and PerCP-labeled anti-mouse IL-4 for Th2 (BioLegend) and FITC-labeled anti-mouse CD4 antibody and PE-labeled anti-mouse IL-17 (BioLegend) for Th17 [30].

#### Enzyme-linked immunoassay

We quantified IL-4 and IL-17 from intestinal mesentery and sIgA and β-defensin from intestinal mucosal tissue using an enzyme-linked immunoassay (Zenix-320 microplate reader) according to the manufacturer’s instructions (Bioassay Technology Laboratory, Shanghai, China) [28].

### Statistical analysis

Data are presented as mean±standard deviation (SD). The differences between groups are analyzed by one-way analysis of variance and Kruskal–Wallis test using SPSS software ver. 23 (IBM, NY, USA). The significance value was set at p<0.05.

## Results

**Figure-1:**
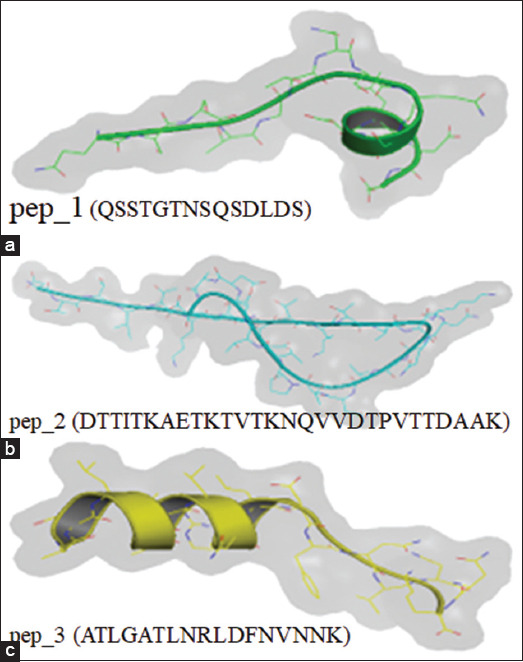
The secondary structure modeling of selected peptide. (a) pep_1 (QSSTGTNSQSDLDS) 14-mer. (b) pep_2 (DTTITKAETKTVTKNQVVDTPVTTDAAK) 28-mer and (c) pep_3 (ATLGATLNRLDFNVNNK) 17-mer.

**Figure-2:**
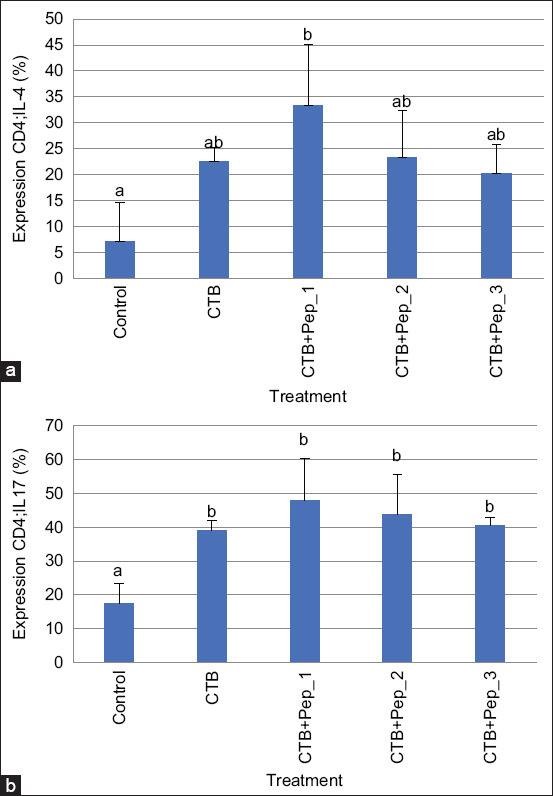
Cellular immune response of intestinal mesenteric tissue of mice following oral immunization was determined by flow cytometry. (a) The significant increase of Th2 expressing CD4-IL4 is significantly higher in immunized groups (b). The significant increase of Th17 expressing CD4-IL17 is significantly higher in immunized groups, especially in CTB+pep_1 group. Data are shown as mean±SD.

**Figure-3:**
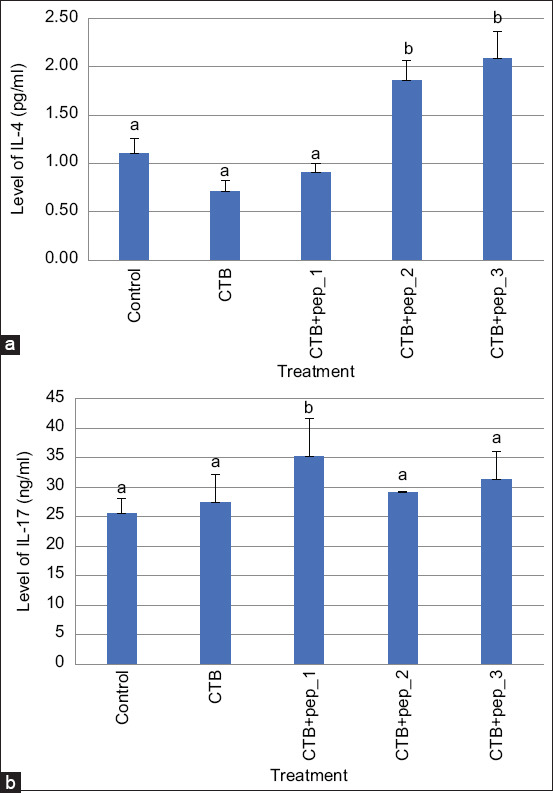
Humoral immune response of intestinal mesenteric tissue of mice following oral immunization was determined by ELISA. (a) The significant increase of IL-4 in CTB_pep_2 and CTB_pep 3 groups (b). The significant increase of IL-17 in CTB+pep_1 group. Data are shown as mean±SD.

**Figure-4:**
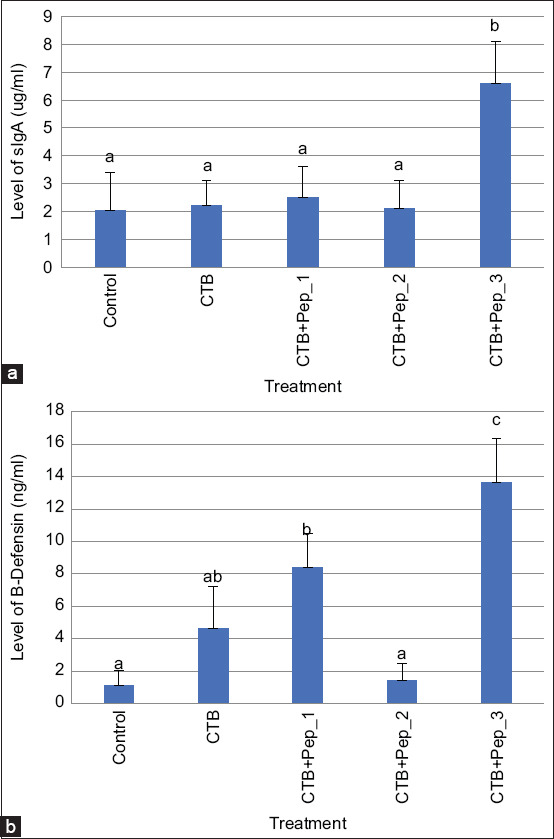
Cellular immune response of intestinal mucosal layer of mice following oral immunization was determined by ELISA. (a) The significant increase of sIgA in CTB_pep_3 group (b). The significant differences of β-defensin level among immunized groups. Data are shown as mean±SD.

Figure-1 shows the secondary structures of the three selected peptides obtained by modeling. Pep_1, Pep_2, and Pep_3, respectively, contain a helix, a coil (thread), and a sheet. Figure-2 shows the cellular immune responses. The immunization groups had significantly higher Th2 and Th17 levels than the control group. The CTB+pep_1 group had the highestTh2 (p=0.019) and Th17 levels (p=0.004). Regarding the humoral immune response, the immunization groups, particularly the CTB+pep_3 group (p=0.000), had increased IL-4 levels. However, only the CTB+pep_1 group (p=0.042) had a significantly higher IL-17 level than the control group (Figure-3). Finally, Figure-4 presents the mucosal immune response results. The CTB+pep_3 group had a significantly elevated sIgA level (p=0.042). Moreover, the immunization groups had higher levels of the natural immune response marker β-defensin (p=0.000).

## Discussion

*S. flexneri* enters the epithelial cells by transiting through M cells and encounters resident macrophages. Pro-inflammatory signals released by macrophages and epithelial cells activate a natural immune response involving natural killer cells and polymorphonuclear cells [31]. *Shigella* relies on the production of effector proteins that contribute to manipulating the infectious process as a specific mechanism to invade host cells [32]. Two types of attachment proteins of *Shigella* are fimbriae/pili proteins and the OMPs. Pili proteins and OMPs are virulence factors for a bacterial colonization that causes infection [33,34]. Therefore, developing OMP epitope-based vaccines against *Shigella* infection are a sensible strategy [22]. We identified the potential of *S. dysenteriae*, *S. flexneri*, *S. sonnei*, and *S. boydii* pili proteins as adhesive proteins that can be used for *Shigellosis* vaccine development [7,9]. The current study revealed the development of an epitope-based vaccine candidate against *Shigella* with good prospects and safety. We utilized three antigenic peptides derived from the 49.8 kDa pili protein subunit of *S. flexneri* and demonstrated that they induced the immune cellular and humoral responses as protection mechanisms against *Shigella*.

Oral vaccination against shigellosis can increase local immune response as linear with the pathogenesis of *Shigella*. In this study, we administered immunizing compounds orally and assessed the mucosal immune response through sIgA and β-defensin, which are essential immune system proteins in enteric infections. Preventing enteric mucosal infections in humans requires IgA secretion. *S. flexneri*-infected patients had significantly more cells secreting circulating antibodies than healthy individuals [35]. Activated CD4+ T cells differentiate into Th1, Th2, Th17, and regulatory T cells [36,37]. Th2 cells produce mediator cytokines such as IL-4, IL-5, and IL-10, which stimulate the production of plasma cell antibodies, including IgA. An intensive increase in IgA production reflects the stimulation of the mucosal defense [38]. Activated Th17 cells differentiate and secrete IL-17 and IL-22. IL-17 increases the secretion of sIgA into the lumen [16]. In addition, IL-17 and IL-22 can help intestinal epithelial cells produce antimicrobial proteins [15]. IL-17 and IL-22 strongly stimulate AMPs secretion by epithelial cells [39].

Based on the mechanism of *Shigella* pathogenesis and immune response, we measured the ability of our peptides to stimulate the immune responses by quantifying Th2, IL-4, and sIgA. Treating mice with 49.8 kDa *S. flexneri* pili protein subunit-based peptides increased the Th2 cellular immune response. The three peptides tested (pep_1, pep_2, and pep_3) were immunogenic. The ability of these peptides to induce IL-4 production by Th2 cells is encouraging for vaccine design [40]. This correlates with the previous studies that reported increased lymphocyte proliferation after vaccination with the rIpaB protein of *S. flexneri* and rGroEL of *Salmonella typhi* [41]. *Salmonell*a secretes effector I (SseI or SrfH) and the flagellin protein FliC, which activates T cells. Inducing the immune response of CD4 T-cells using the epitopes of these proteins protects against infection [42,43]. The proliferation of Th2 cells increases the levels of IL-4, a cytokine that plays a key role in numerous biological activities [44].

The cytokine IL-4 is part of the humoral immune response. The CTB+pep_2 and CTB+ pep_3 groups had higher IL-4 levels than the control group. A previous study reported a similar IL-4 production after administering a peptide derived from the EpiMix protein of *S. flexneri* [45]. Another study revealed that the recombinant chimeric protein rMESF of *S. flexneri* significantly increased IL-4 levels through all tested application routes; intranasal, intravenous, intramuscular, and subcutaneous [46].

Mucosal antibody production is proportional to Th2 and IL-4 levels. Th2 cells produce IL-4 cells, activating sIgA production, facilitating B cells activation and antibody release. B cells in the lamina propria produce IgA, protecting the mucosal tissue and neutralizing microbes before invading the mucosal lumen. sIgA acts the outer bacterial membrane and inhibits the bacterial attachment to mucosal surfaces [35,47]. The increase in sIgA levels in this study suggested that our vaccine candidates stimulated the mucosal immune responses. The vaccine using *S. flexneri* rMESF multiepitope protein increases sIgA-specific antibodies in the mucus layer and system wide [46]. EpiMix, *S. flexneri* protein fragment, produces a specific sIgA mucosal immune response from immunized animal fecal samples [45]. The presence of pathogen-specific sIgA is important to prevent the infection of mucosal tissues. IgA is an immunoglobulin essential *Shigella* infection and reinfection prevention and treatment. Therefore, IgA production inducing antibodies are the primary protection mechanism against *Shigella* infection [48].

Our study assessed the immune response through Th17 cells, IL-17, and β-defensin. Th17 cells produce the main cytokines, namely, IL-17 and IL-22. In addition, Th17 cells stimulate the production of antimicrobial substances called defensins that function like locally produced endogenous antibiotics [37]. Our peptides successfully activated the Th17 cellular immune response. The ability of these peptides to activate Th17 cells demonstrates their strong potential as shigellosis vaccine candidates. Numerous scientific reports show that Th17 cells and IL-17 can induce the production of antimicrobial cytokines and proteins that contribute to the host’s defense system against intestinal pathogens. IL-17 increases sIgA secretion into the lumen as well [16]. *Shigella* infection induces IL-17 and IL-22 by Th17 cells, increasing the host’s defense against bacteria and fungi [15].

IL-17 is a cytokine mainly produced by Th17 cells involved in the protection against pathogens in mucosal tissues [49,50]. Therefore, an increase in IL-17 levels indicates the efficacy of a vaccine [51], and pep_1, pep_2, and pep_3 increased the IL-17 levels. Our results are consistent with the previous studies [46]. IL-17 and IL-22 can stimulate the production of antimicrobial proteins by intestinal epithelial cells [15]. Antimicrobial proteins are a gastrointestinal mucosal defense against pathogens [52].

AMPs are part of the natural humoral immune systems of the innate immune response against various pathogenic organisms. They govern the regulatory signals for the innate and adaptive immune systems to eliminate pathogens [17]. Most mammals express AMPs from the defensin and cathelicidin families [53]. Defensins play an important role in biological processes because of their antimicrobial and immunomodulatory activities [17]. β-defensin has a positive charge, effective against negatively charged bacteria [54]. Our results showed that our peptides increased β-defensin levels. The presence of β-defensins is crucial because they can attract inflammatory cells such as neutrophils, T cells, and macrophages [55].

Despite the high efficacy of peptide-based oral vaccines, the utilization of peptides as antigens for oral vaccine development faces several limitations, such as proteolysis degradation. However, using delivery systems, co-administrating protease inhibitors can enhance the effect of vaccine candidates [56,57]. Therefore, we aim to enhance the efficacy of our peptide candidate and optimize antigen delivery systems in further studies.

## Conclusion

It can be concluded that peptides derived from the 49.8 kDa *S. flexneri* pili protein subunit as prospective shigellosis vaccines by demonstrating their ability to induce the mucosal immune response. The pep_3 peptide sequence was particularly efficient. Further studies involving peptide delivery systems and neutralization examination through bacterial colony growth inhibition are necessary to realize the potential of the selected peptide.

## Authors’ Contributions

SRP and HS: Conceptualization. KA, ATE and DYNH: Designed the study and research investigation. KA and SRP: Performed data analysis, data interpretation, and drafted the manuscript. All author have read and approved the final manuscript.
